# Case report: Adolescent breast hypertrophy with a tendency toward phyllodes tumor formation: first reported case in China

**DOI:** 10.3389/fonc.2025.1652446

**Published:** 2025-10-15

**Authors:** Ruiheng Liao, Yang Xiao, Yujing Chang, Zhang Zhang, Yange Zhang

**Affiliations:** ^1^ Department of Plastic and Burn Surgery, West China Hospital of Sichuan University, Chengdu, China; ^2^ Department of Pathology, West China Hospital of Sichuan University, Chengdu, China

**Keywords:** adolescent breast hypertrophy, reduction mammoplasty, phyllodes tumor, pathology, breast tumor

## Abstract

Adolescent breast hypertrophy is a rare and persistent condition that affects peripubertal females both physiologically and psychologically. We present a case of a 14-year-old patient with bilateral giant breast enlargement who underwent a successful reduction mammoplasty of 16.5% Body Weight. The right breast exhibited focal interstitial proliferation myofibroblasts, suggesting a potential risk for phyllodes tumor development. This finding represents a novel histopathological association, unreported in the literature and first documented in China.

## 1 Introduction

Adolescent breast hypertrophy, also known as juvenile macromastia or virgin breast hypertrophy, is a benign condition characterized by rapid, disproportionate unilateral or bilateral breast enlargement during puberty (1). Its precise etiology remains unclear (2). Hormonal assays are typically unremarkable (3–5). Early intervention may be beneficial for normal social activities and mental health in adolescence (6, 7). This report describes a case of a 14-year-old Chinese female with adolescent breast hypertrophy who underwent surgical intervention. Notably, postoperative pathology suggested features indicative of phyllodes tumor transformation.

## 2 Case report

**Figure 1:**
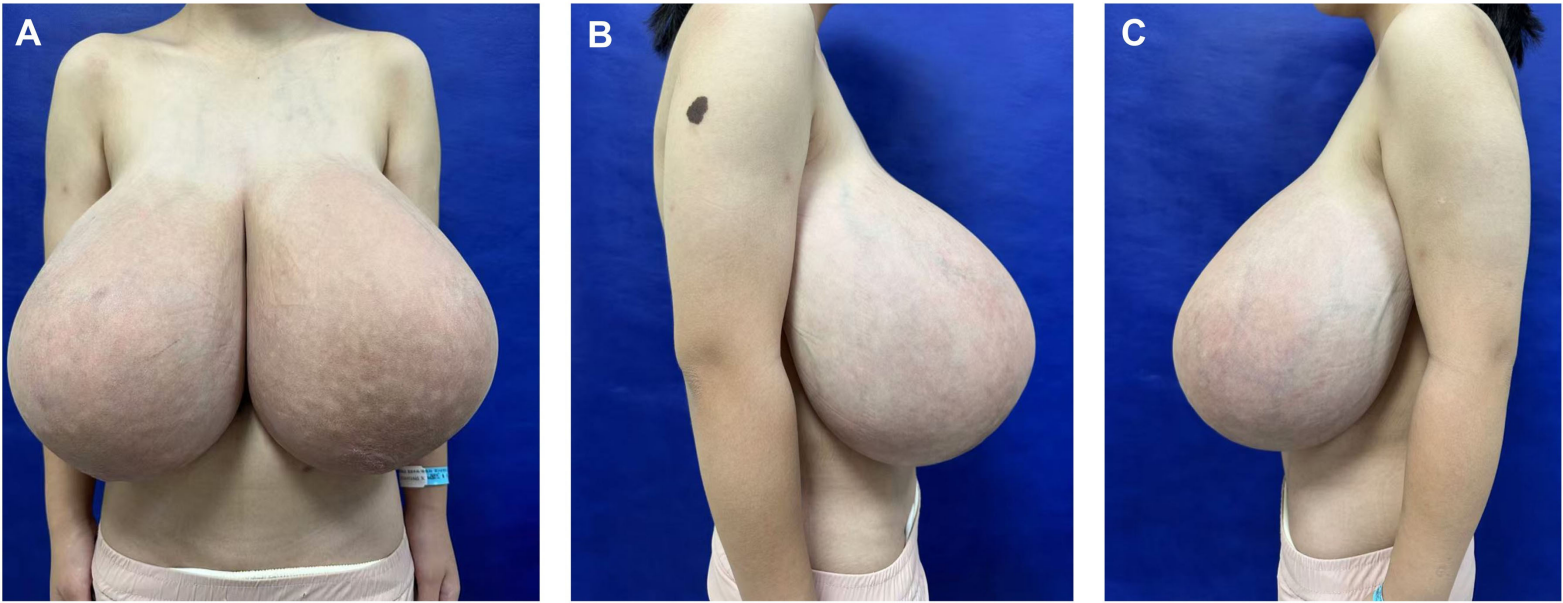
Preoperative frontal **(A)** and lateral **(B, C)** views of a patient with adolescent macromastia.

A 14-year-old female patient presented to our Plastic and Reconstructive Surgery Department with excessive breast volume and ptosis. The patient’s height was 155 cm, preoperative weight was 59 kg, and body mass index (BMI) was 24 kg/m². Progressive bilateral breast enlargement over two postpubertal years was accompanied by prominent superficial venous engorgement. The patient’s breasts were basically symmetrical on both sides, with the right breast sagging 10 cm below the rib cage and the left sagging 9 cm below the rib cage (**Figure 1**). Excessive breast volume precluded accurate volumetric MRI assessment. Preoperatively she underwent a puncture biopsy of the right breast mass, which indicated mesenchymal hyperplasia accompanied by normal-type ductal epithelial hyperplasia. No contributory family history, pharmacotherapy, or gonadotropin axis abnormalities were identified. Breast ultrasonography demonstrated multiple bilateral solid breast lesions suggestive of fibrocystic hyperplasia. Therefore, we can temporarily rule out abnormal breast enlargement caused by endocrine disorders or local breast lesions such as tumors or inflammations.

**Figure 2:**
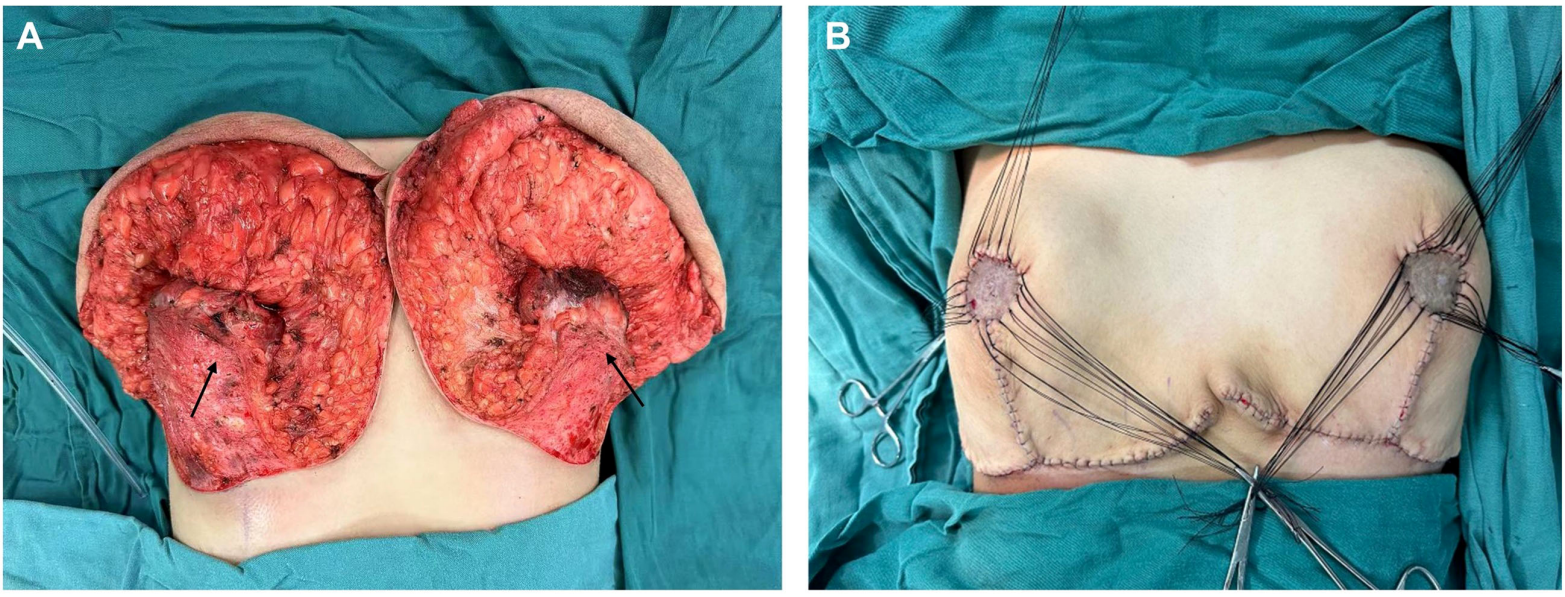
The illustrations show the breast gland that was preserved during the surgery. (The arrow in the figure indicates the pedicle preserved during surgery).

**Figure 3:**
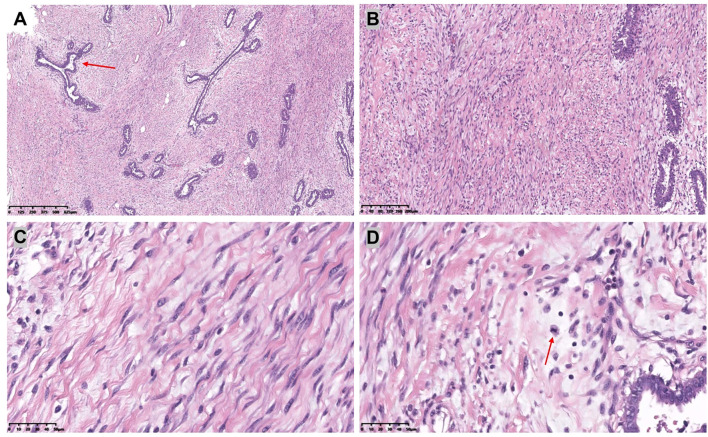
[**(A):** H&E,×4, **(B):** H&E,×10] The bilateral mammary glands were composed of dilated ducts and hyperplastic epithelium. There was a proliferation of interstitial fibrous tissue and myofibroblasts.[**(C):** H&E,×40] Myofibroblasts are blazingly proliferative. [**(D):** H&E,×40] The interstitium showed prominent mitotic activity.

Given the rapid increase in breast volume and the patient’s strong desire for surgical intervention, medical therapy was no longer considered the preferred approach. Furthermore, evidence suggests that in cases involving long pedicles (>10 cm mobilization), large reductions (>2000 g), or severe ptosis, consideration should be given to free nipple–areola complex grafting (8). After thorough preoperative communication with the patient and her family members, the inverted-T incision with inferior pedicle technique and free nipple-areola complex (NAC) graft were ultimately chosen as the operative approach. The distances from the right and left NACs to the inframammary fold (IMF) were 16 cm and 15 cm, respectively. The neo-nipple areolar complex (neo-NAC) on both breasts was positioned 26 cm from the sternal notch. The total amount of breast tissue removed during the operation was 9.6 kg, with 4.7 kg excised from the left side and 4.9 kg from the right (**Figure 2**). Histopathological analysis revealed bilateral hyperplasia of interstitial fibrous tissue and myofibroblasts, accompanied by collagenization. The right breast additionally exhibited nuclear fission and pronounced focal hyperplasia of interstitial myofibroblasts, indicating a tendency toward phyllodes formation (**Figure 3**). Immunohistochemical staining of the right breast lesion showed: Desmin (–), SMA (+), CD10 (+), CD34 (+), S-100 (–), SOX10 (–), EMA (–), and Ki67 positivity (10%-15%). Postoperative pathological examination revealed prominent focal interstitial proliferation with mitotic figures. The immunophenotype supported a stromal origin, excluding epithelial or neurogenic lesions. The stroma lacked a typical leaf-like architecture, and based on these findings, the diagnosis was bilateral mammary hypertrophy with a phyllodes-like tendency in the right breast. Given these findings suggesting malignant potential, we recommended close follow-up with biannual postoperative breast imaging (ultrasound and MRI) to monitor recurrence or malignancy.

**Figure 4:**
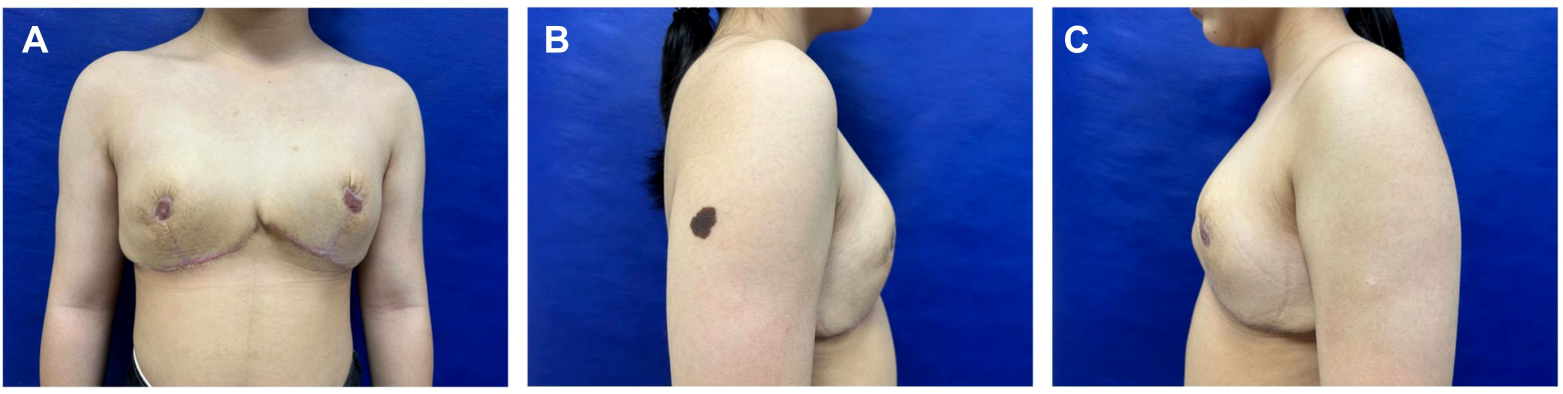
Postoperative frontal **(A)** and lateral **(B, C)** views 6 months later.

**Figure 5:**
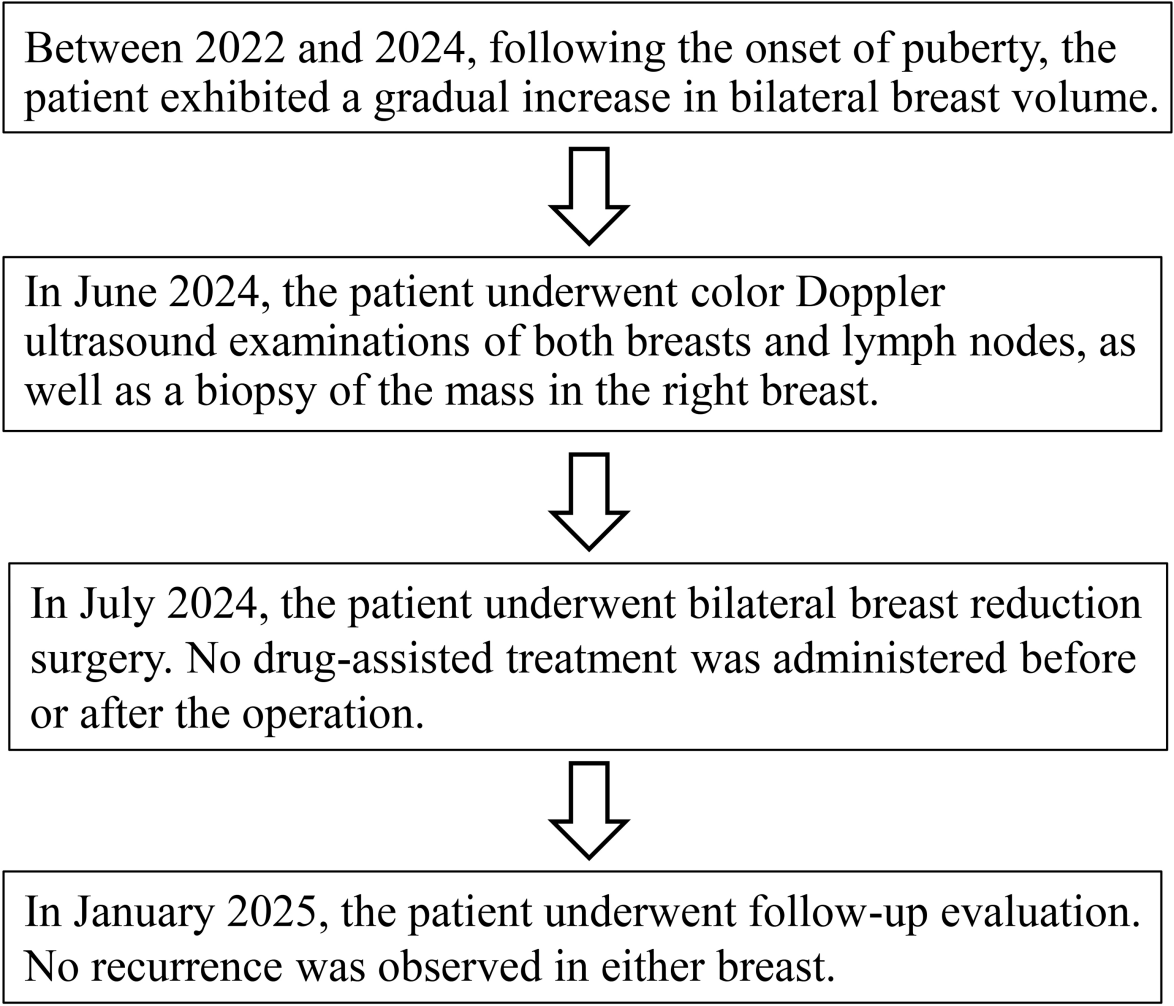
Overview of the patient treatment process flowchart.

The patient was discharged on postoperative day 7 without medication. At 6-month follow-up, she reported satisfaction with breast aesthetics, and there were no signs of recurrence. (**Figure 4**). Postoperative magnetic resonance imaging (MRI) revealed left and right breast volumes of 632 cm³ and 605 cm³, respectively. Bilateral breast ultrasonography revealed no significant abnormalities (**Figure 5**). Her emotional state and social participation improved significantly—she actively joined the school badminton club, no longer experienced anxiety when choosing clothing, and reported resolution of the sleep disturbance associated with supine positioning prior to surgery. These subjective improvements were consistent with the objective therapeutic outcomes, further supporting the value of breast reduction surgery in adolescent patients with macromastia. Given the patient’s adolescent stage, long-term surveillance is recommended every 6–12 months thereafter.

## 3 Discussion

Adolescent breast hypertrophy is characterized by the excessive development of breast tissue in adolescent females, resulting in disproportionate breast volume relative to torso size (1). This rapid breast growth can lead to skin stretching, loss of areolar definition, and even ulceration (9, 10). Besides the symptoms like back and neck pain, the patient’s primary grievances stemmed from social awkwardness and her avoidance of social engagements, which were caused by her exceptionally large breast size. The etiology and pathogenesis of this condition are complex, involving various factors such as hormone levels, genetic predispositions, receptor sensitivity, and environmental influences (2, 11–14). Notably, significant unilateral or bilateral enlargement can occur despite normal gonadal hormone levels, suggesting idiopathic end-organ hypersensitivity— manifested as elevated estrogen sensitivity or progesterone receptor upregulation (2, 15, 16). This patient’s endocrinology profile were normal, and there was no history of related medication use. The condition may be associated with changes at the genetic or molecular level. Currently, there is no published data clarifying the prevalence of adolescent breast hypertrophy. With the rising health awareness and environmental shifts, more individuals are seeking specialized medical advice and treatment for adolescent breast hypertrophy in hospitals (15). Therapeutic strategies comprise medical and surgical interventions. Pharmacotherapy (e.g., tamoxifen, bromocriptine) demonstrates limited efficacy in established macromastia but may adjunctively prevent postoperative recurrence (4, 5, 17). For this specific patient, given the limited efficacy of pharmacotherapy and the risks associated with adverse drug reactions, surgical treatment alone was sufficient to achieve the therapeutic goals, eliminating the need for additional drug assistance. Since the patient’s breast volume was still increasing, the inverted-T incision with inferior pedicle technique and free nipple-areola complex (NAC) graft was ultimately selected to optimize the removal of glandular tissue and minimize the risk of recurrence after surgery, while also considering the shape and volume of the breast. Compared with pedicled reductions, staged procedures, or skin-sparing resections, this technique enables more effective and complete management of enlarged glands within a shorter treatment course, while simultaneously resolving the issues of skin excess and residual hyperplasia (18, 19). Besides, Fiumara et al. provided statistical evidence suggesting that the use of free nipple–areola complex grafting is associated with a lower recurrence rate compared with the pedicled technique (20).

Based on the H&E staining and the immunohistochemical results provided, the pathological examination of this case revealed phyllodes tumor formation predisposition in the right breast. This pathological type has not been reported previously and expands the clinicopathological spectrum of the disease. This type of macromastia indicates that we should consider early assessment, intervention, or surgical treatment. Phyllodes tumors are known for their high recurrence rate. Phyllodes tumors can be classified into three categories: benign, borderline, and malignant. Nuclear division count is one of the core criteria for grading of nodular tumors. Its combined assessment with the Ki-67 index can significantly improve the diagnostic accuracy (21, 22). Benign phyllodes tumors are the most common and exhibit histological and clinical features similar to those of fibroadenomas. Higher-grade phyllodes tumors correlate with increased risk of recurrence or metastasis. The similar histologic features of phyllodes tumors and fibroadenomas warrant careful evaluation for the potential progression of fibroadenomas (23). Currently, the standard treatment for phyllodes tumors involves surgical excision with clear margins, often in conjunction with breast-conserving surgery and is closely monitored during postoperative follow-up (24).

Adolescent patients face recurrence risks from ongoing breast development or pregnancy-related hormonal shifts (4, 13, 25, 26). Consequently, we prioritized reduction mammoplasty as initial therapy, reserving mastectomy for recurrence. There were no signs of recurrence during 6-month follow-up period. The girl was pleased although the aesthetic result was only acceptable from an objective point of view. However, this case also has some limitations, including a relatively short follow-up period and potential diagnostic uncertainties.

## 4 Conclusion

Surgical intervention remains the primary treatment option for adolescent breast hypertrophy. Medical therapy demonstrates promising potential for disease control and recurrence risk reduction. However, further research is warranted.

## Data Availability

The raw data supporting the conclusions of this article will be made available by the authors, without undue reservation.
